# Functional in-vitro evaluation of the non-specific effects of BCG vaccination in a randomised controlled clinical study

**DOI:** 10.1038/s41598-022-11748-x

**Published:** 2022-05-12

**Authors:** Morven Wilkie, Rachel Tanner, Daniel Wright, Raquel Lopez Ramon, Julia Beglov, Michael Riste, Julia L. Marshall, Stephanie A. Harris, Paulo J. G. Bettencourt, Ali Hamidi, Pauline M. van Diemen, Paul Moss, Iman Satti, David Wyllie, Helen McShane

**Affiliations:** 1 Jenner Institute, Nuffield Department of Medicine, University of Oxford, Oxford, OX3 7DQ UK; 2 Department of Infection and Tropical Medicine, Birmingham Heartlands Hospital, Birmingham, B9 5SS UK; 3 Present Address: Division of Cardiovascular Medicine, Radcliffe Department of Medicine and British Heart Foundation Centre of Research Excellence Oxford, University of Oxford, Oxford, OX3 9DU UK; 4 Present Address: Department of Infection and Tropical Medicine, North Manchester General Hospital, Manchester, M8 5RB UK; 5 Present Address: Faculty of Medicine and Center for Interdisciplinary Research in Health, Catholic University of Portugal, 1649-023 Lisbon, Portugal; 6 Present Address: Chelsea and Westminster Hospital, London, SW10 9NH UK; 7 Present Address: Animal and Plant Health Agency (APHA), Virology, APHA-Weybridge, New Haw, Addlestone, KT15 3NB UK

**Keywords:** Immunology, Microbiology, Medical research

## Abstract

Bacille Calmette-Guérin (BCG), the only currently licenced tuberculosis vaccine, may exert beneficial non-specific effects (NSE) in reducing infant mortality. We conducted a randomised controlled clinical study in healthy UK adults to evaluate potential NSE using functional in-vitro growth inhibition assays (GIAs) as a surrogate of protection from four bacteria implicated in infant mortality. Volunteers were randomised to receive BCG intradermally (*n* = 27) or to be unvaccinated (*n* = 8) and were followed up for 84 days; laboratory staff were blinded until completion of the final visit. Using GIAs based on peripheral blood mononuclear cells, we observed a significant reduction in the growth of the Gram-negative bacteria *Escherichia coli* and *Klebsiella pneumonia* following BCG vaccination, but no effect for the Gram-positive bacteria *Staphylococcus aureus* and *Streptococcus agalactiae*. There was a modest association between *S. aureus* nasal carriage and growth of *S. aureus* in the GIA. Our findings support a causal link between BCG vaccination and improved ability to control growth of heterologous bacteria. Unbiased assays such as GIAs are potentially useful tools for the assessment of non-specific as well as specific effects of TB vaccines. This study was funded by the Bill and Melinda Gates Foundation and registered with ClinicalTrials.gov (NCT02380508, 05/03/2015; completed).

## Introduction

Introduced a century ago, Bacille Calmette-Guérin (BCG) remains the only licensed tuberculosis (TB) vaccine. BCG vaccination confers high levels of protection against severe TB disease in infants but fails to protect consistently against adult pulmonary disease^1,2^. Development of a more efficacious vaccine is vital to achieve the World Health Organisation (WHO) target of ending the global TB epidemic by 2035. It has been proposed for over 50 years that BCG may exert beneficial ‘non-specific’ effects (NSE) on the immune system^3^. Randomised and observational studies in low-income countries indicate lower all-cause mortality rates for BCG-vaccinated neonates; most consistently associated with a reduction in cases of sepsis, respiratory infection and fever^4–10^. Furthermore, a randomised controlled trial found that BCG vaccination protects against non-tuberculous infectious disease during the neonatal period in Uganda^11^ and a trial of BCG revaccination reported a reduced rate of upper respiratory tract infections^12^. Some studies have suggested more marked protective NSE of BCG in girls than boys^13–16^, although two systematic reviews did not find a sex-differential component^17,18^.

The advent of the COVID-19 pandemic has triggered a resurgence of interest in NSE of BCG vaccination. Several early ecological and epidemiological studies suggested that countries with a BCG vaccination program had reduced COVID-19 infections, severity and/or mortality, but have been widely criticised for confounders^19^. More than 20 clinical trials are now assessing the efficacy of BCG vaccination against COVID-19. The mechanism(s) underlying BCG-mediated NSE have not been fully elucidated but may involve cross-reactive/antigen-independent heterologous lymphocyte responses and/or ‘trained’ innate immune memory mediated by epigenetic changes^20–25^.

While evidence in favour of vaccine-induced NSE is arguably strongest for BCG, study outcomes have in some cases been conflicting or confounded, and the clinical relevance of NSE has been subject to debate^26–29^. The WHO Strategic Group of Experts on Immunisation has concluded that the NSE of BCG vaccination on all-cause mortality warrants further research^30^. It is essential that the cogency of this effect is clarified because: (a) potential BCG replacement TB vaccines must be demonstrated to be non-inferior in this regard, in addition to being safe and efficacious against TB; (b) NSE could be exploited, mimicked or augmented using novel vaccines; (c) discontinuing BCG vaccination programmes due to a decline in TB prevalence may have detrimental knock-on effects; and (d) BCG vaccination may have value in the early control of future emerging pathogens.

**Figure 1:**
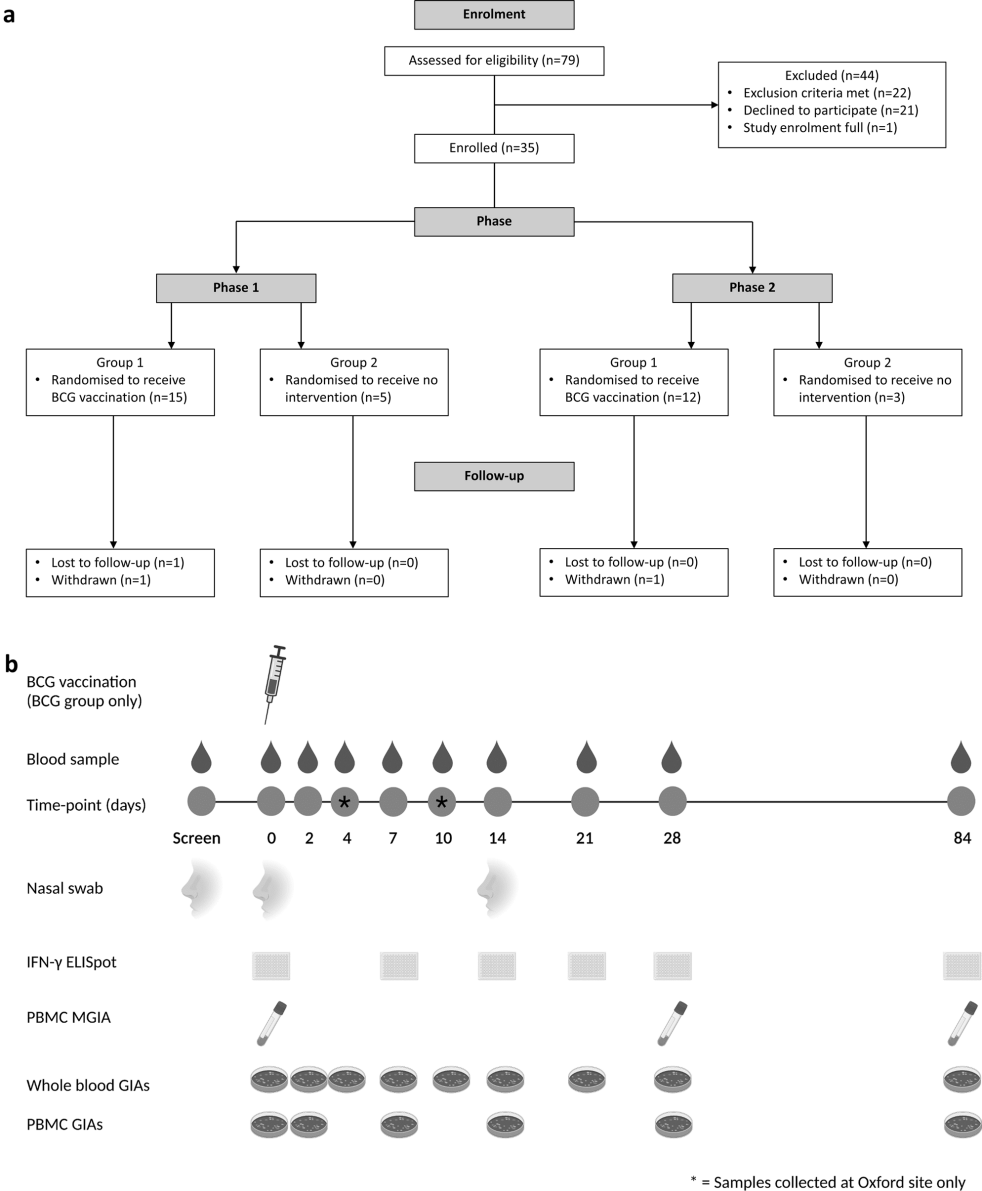
Consort diagram of volunteer recruitment and study schedule. Volunteers (see Table 1 for demographics) were enrolled in two phases and randomised to receive either BCG vaccination or no intervention (**a**). Volunteers had blood samples collected at screening and days 0 (baseline), 2, 4, 7, 10, 14, 21, 28 and 84. Volunteers from Birmingham followed the same schedule with the exception of follow-up visits on days 4 and 10 which were omitted for logistical reasons. Nasal swabs for determination of *S. aureus* carriage status were taken at screening and days 0 and 14 (**b**).

We conducted a randomised controlled clinical study of BCG vaccination in healthy UK adults to investigate the purported NSE of BCG vaccination on human immunity to heterologous pathogens using functional in-vitro growth inhibition assays (GIAs) as a surrogate of protection. We assessed the ability of whole blood or cells, taken from volunteers before and after BCG vaccination, to control the growth of four different bacteria associated with neonatal and/or childhood mortality in developing countries: *Staphylococcus aureus (S. aureus)*, *Streptococcus agalactiae (S. agalactiae), Escherichia coli (E. coli)* and *Klebsiella pneumoniae (K. pneumoniae)*^31–34^*.* We hypothesised that improved control of these bacteria would be observed following BCG vaccination. As a secondary aim, we explored the association between *S. aureus* nasal carriage and ability to control bacterial growth *in-vitro* as this may represent a potential confounder to GIA outcomes (Fig. 1).

## Results

### Specific responses to BCG vaccination were as expected

#### PPD-specific IFN-γ ELISpot responses were increased following BCG vaccination

**Figure 2:**
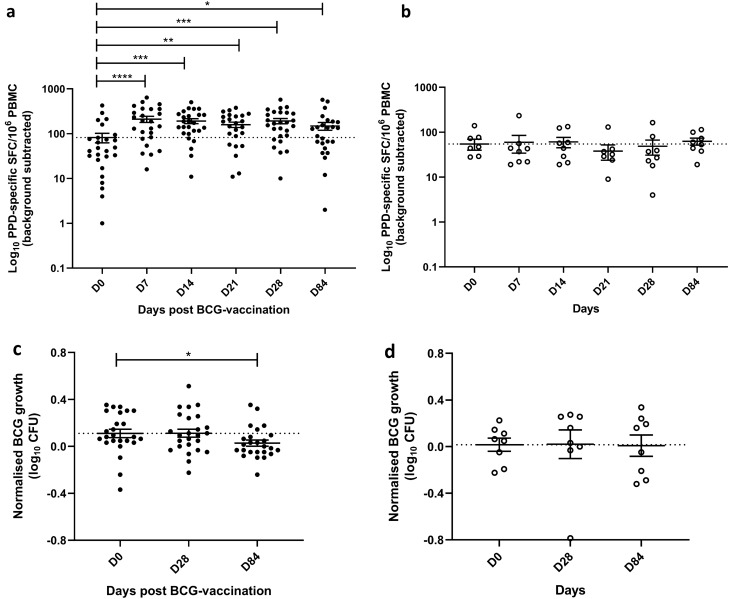
PPD-specific IFN-γ ELISpot responses and control of mycobacterial growth in the direct PBMC MGIA are enhanced following BCG vaccination. Samples were taken from volunteers enrolled into phases 1 and 2 combined. Healthy UK adults were randomised to receive BCG vaccination (*n* = 27) or to be unvaccinated controls (*n* = 8). PPD-specific IFN-γ ELISpot responses were measured at baseline and days 7, 14, 21, 28 and 84 in volunteers who received BCG vaccination (**a**) and those who were unvaccinated controls (**b**). The direct PBMC MGIA was conducted on cells and serum taken at baseline and days 28 and 84 from volunteers who received BCG vaccination (**c**) and those who were unvaccinated controls (**d**). Bars represent the mean values with the standard error of the mean (SEM); dotted lines indicate the baseline mean. For A–B, Wilcoxon tests were performed of each time-point vs. baseline. For C–D, a paired t-test was performed of each time-point vs. baseline. *Indicates a *p*-value of < 0.05, **indicates a *p*-values of < 0.005 and ***indicates a *p*-value of < 0.001. SFC = spot-forming cells.

Specific responses to BCG vaccination were assessed to confirm vaccine ‘take’. In the volunteers that received BCG vaccination, ex-vivo IFN-γ ELISpot responses to PPD were significantly increased at 7, 14, 21, 28 and 84 days post-vaccination compared with baseline (*p* < 0.0001, *p* = 0.0004, *p* = 0.008, *p* = 0.0001 and *p* = 0.03 respectively, Fig. 2a). Following correction for multiple comparisons, significance remained at 7, 14 and 28 days (*p* < 0.0001, *p* = 0.0003 and *p* < 0.0001 respectively) with a median count of 44.5 PPD-responsive spot forming cells (SFC) per million PBMC at baseline and 167, 157.5 and 168 SFC at 7, 14 and 28 days, respectively. There was no change in response over time in the unvaccinated volunteers (Fig. 2b).

#### Control of mycobacterial growth in the direct PBMC MGIA was enhanced following BCG vaccination

Mycobacterial growth in the direct PBMC MGIA was significantly reduced at day 84 following BCG vaccination compared with baseline (*p* = 0.04, Fig. 2c), which remained significant after correction for multiple comparisons (*p* = 0.04). There was no change in response over time in the unvaccinated volunteers (Fig. 2d) and no significant differences between vaccinated and unvaccinated volunteers at any of the time-points.

### Bacterial GIAs detected non-specific effects of BCG vaccination

#### Whole blood GIAs were unsuitable for the intended investigation

Due to logistical limitations, *S. aureus* and *E. coli* GIAs were performed on whole blood taken from volunteers enrolled into phase 1, while *K. pneumoniae* and *S. agalactiae* GIAs were performed on whole blood taken from volunteers enrolled into phase 2. Although we observed a significant reduction in bacterial growth of *S. aureus* and *K. pneumoniae* at some time-points following BCG vaccination (Figure S1A and G respectively), there was a similar effect in the unvaccinated control group (Figure S1B and S1H). We did not observe such differences with *S. agalactiae* or *E. coli,* but noted considerable heterogeneity between participants, particularly for *E. coli* (Figure S1C, D, E, F)*.*

There was a reduction in haemoglobin (Hb) over time in both the BCG-vaccinated and unvaccinated volunteers which may have confounded bacterial growth in the whole blood GIAs. In the vaccinated group, Hb decreased significantly at days 7, 10, 14, 21 and 28 relative to baseline (*p* < 0.0001, *p* = 0.003, *p* < 0.0001, *p* < 0.0001, *p* < 0.0001 respectively, Figure S2A). These differences remained significant after correction for multiple comparisons. In the control group, Hb decreased significantly at days 7 and 10 compared to baseline (*p* = 0.049 and *p* = 0.049 respectively, Figure S2B). We concluded that whole blood GIAs were unsuitable for the longitudinal investigation planned.

#### PBMC GIAs showed reduced growth of gram-negative bacteria following BCG vaccination

**Figure 3:**
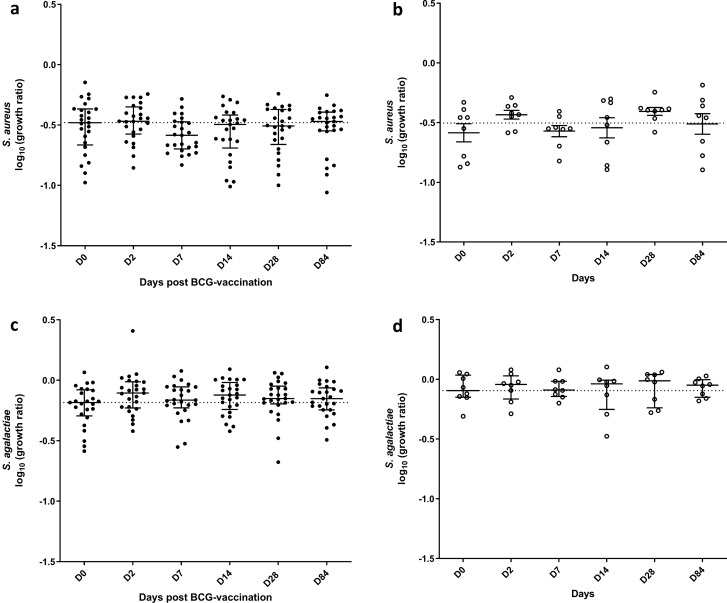
No effect of BCG vaccination on growth of Gram-positive bacteria in the PBMC GIA. Samples were taken from volunteers enrolled into phases 1 and 2 combined. Healthy UK adults were randomised to receive BCG vaccination (*n* = 27) or to be unvaccinated controls (*n* = 8). PBMC bacterial GIAs were conducted on samples taken at baseline and days 2, 7, 14, 28 and 84 following BCG vaccination (closed circles) and at the same time-points in unvaccinated control individuals (open circles). PBMC and autologous serum were co-cultured with *S. aureus* (**a, b**) or *S. agalactiae* (**c, d**) for 1 h after which time cells were lysed and bacteria quantified by plating on solid blood agar. Bars represent the median values with the interquartile range (IQR); dotted lines indicate the baseline median.

**Figure 4:**
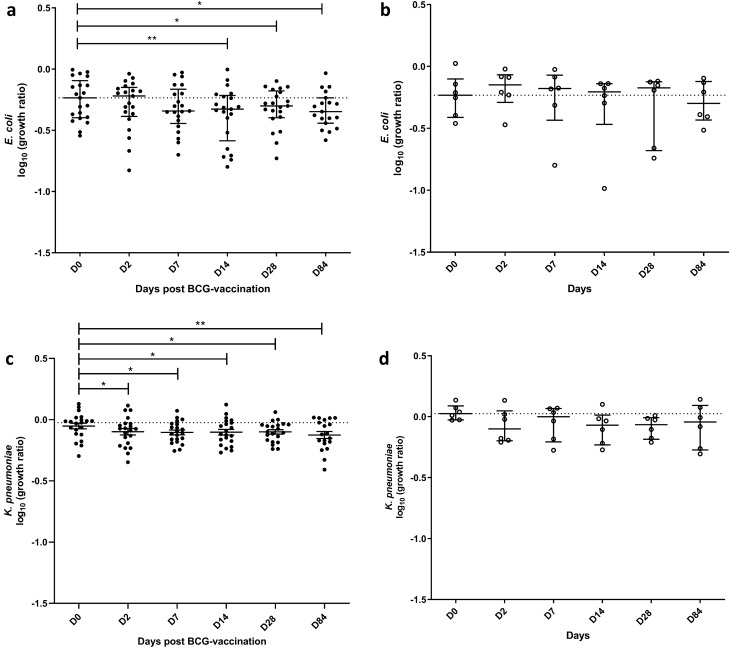
Significant effect of BCG vaccination on growth of Gram-negative bacteria in the PBMC GIA. Samples were taken from volunteers enrolled into phases 1 and 2 combined. Healthy UK adults were randomised to receive BCG vaccination (*n* = 27) or to be unvaccinated controls (*n* = 8). PBMC bacterial GIAs were conducted on samples taken at baseline and days 2, 7, 14, 28 and 84 following BCG vaccination (closed circles) and at the same time-points in unvaccinated control individuals (open circles). PBMC and autologous serum were co-cultured with *E. coli* (**a, b**) or *K. pneumoniae* (**c, d**) for 1 h after which time cells were lysed and bacteria quantified by plating on solid blood agar. Bars represent the median values with the interquartile range (IQR); dotted lines indicate the baseline median. Paired t-tests were performed of each time-point vs. baseline, where *indicates a *p*-value of < 0.05 and **indicates a *p*-value of < 0.005.

We therefore developed an alternative assay. GIAs were performed on cryopreserved PBMC taken from volunteers enrolled into both phase 1 and phase 2 (*n* = 27 BCG-vaccinated and *n* = 8 controls). We did not detect changes in growth of the two Gram-positive bacteria tested, *S. aureus* (Fig. 3a) and *S. agalactiae* (Fig. 3c). By contrast, growth of the two Gram-negative bacteria tested was reduced following BCG vaccination. *E. coli* growth was reduced at days 14, 28 and 84 (*p* = 0.003, *p* = 0.03, *p* = 0.04 respectively, Fig. 4a); following correction for multiple comparisons this remained significant at day 14 (*p* = 0.005). *K. pneumoniae* growth was reduced at days 2, 7, 14, 28 and 84 (*p* = 0.04, *p* = 0.03, *p* = 0.02, *p* = 0.01 and *p* = 0.007 respectively, Fig. 4c); following correction for multiple comparisons this remained significant at days 28 and 84 (*p* = 0.048 and *p* = 0.034 respectively). In the unvaccinated control group, there were no changes over time in the growth of any of the bacteria (Fig. 3b, d, 4b, d), and there were no significant differences between vaccinated and unvaccinated volunteers at any of the time-points. There was no difference in the growth of *S. aureus*, *E. coli* or *K. pneumoniae* between males and females, but there was a modest reduction in growth of *S. agalactiae* in females compared with males at 2 days post-BCG vaccination and a similar but non-significant trend at day 84 (*p* = 0.03 and *p* = 0.09 respectively, Table S1).

**Table 1:** Volunteer baseline demographics.

	BCG (*n* = 27)	Control (*n* = 8)
**Age**		
Mean, years (range)	27 (18–42)	31 (20–45)
**Gender**		
Female, *n* (%)	20 (74)	4 (50)
**Place of birth**		
UK, *n* (%)	23 (85)	4 (50)
**BMI**		
Mean BMI (range)	27 (19–40)	23 (21–33)
**Smoker**		
Yes, *n* (%)	10 (37)	1 (13)
**Alcohol**		
Yes, *n* (%)	21 (78)	7 (88)
< 14 units/week	16 (59)	6 (75)

Absolute numbers or mean values are indicated with range or percentage in brackets.

### *Staphylococcus aureus* carriage status was associated with *S. aureus* growth in the GIA

The prevalence of *S. aureus* positivity was between 26 and 37% at each of the three sampling time-points. Individuals were considered persistent carriers if two or more consecutive cultures were positive (*n* = 9, 26%), intermittent carriers if one or more non-consecutive cultures were positive (*n* = 7, 20%) or non-carriers if all 3 cultures were negative (*n* = 19, 54%). Full results are shown in Table 2.

**Table 2:** Nasal swab results for *S. aureus* carriage status.

S	D0	D14	Colonisation status
−	+	+	Persistent carrier
−	−	−	Non-carrier
−	−	−	Non-carrier
−	−	−	Non-carrier
−	−	−	Non-carrier
+	−	+	Intermittent carrier
+	−	−	Intermittent carrier
−	−	−	Non-carrier
−	+	+	Persistent carrier
−	−	−	Non-carrier
−	+	+	Persistent carrier
+	+	+	Persistent carrier
−	−	−	Non-carrier
+	−	+	Intermittent carrier
−	−	−	Non-carrier
−	−	−	Non-carrier
+	−	−	Intermittent carrier
−	−	−	Non-carrier
−	−	−	Non-carrier
+	−	+	Intermittent carrier
−	−	−	Non-carrier
−	−	−	Non-carrier
+	+	+	Persistent carrier
−	−	+	Intermittent carrier
−	−	+	Intermittent carrier
−	−	−	Non-carrier
−	−	−	Non-carrier
−	+	+	Persistent carrier
−	+	+	Persistent carrier
−	−	−	Non-carrier
−	−	−	Non-carrier
−	+	+	Persistent carrier
−	+	+	Persistent carrier
−	−	−	Non-carrier
−	−	−	Non-carrier
		Total	46% Carriers (*n* = 16)

The anterior nares of all volunteers (*n* = 35) were sampled at baseline and days 7 and 14. Individuals were considered persistent carriers if two or more consecutive cultures were positive, intermittent carriers if one or more non-consecutive cultures were positive or non-carriers if all 3 cultures were negative.

**Figure 5:**
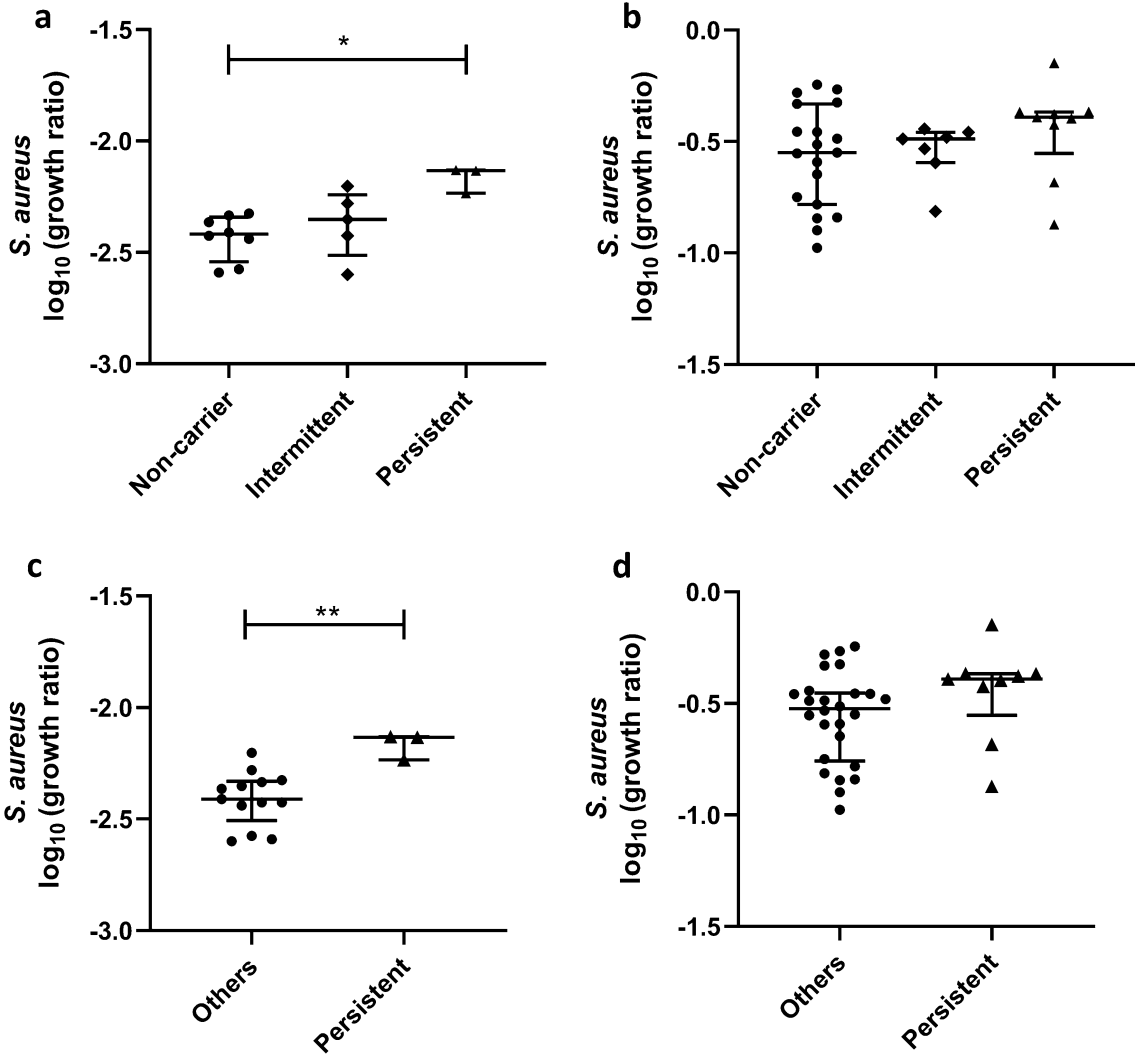
*S. aureus* growth in the GIA stratified by *S.aureus* carriage status. The anterior nares of all volunteers were sampled at screening and days 0 and 14. Individuals were considered ‘persistent’ carriers if two or more consecutive cultures were positive, ‘intermittent’ carriers if one or more non-consecutive cultures were positive or ‘non-carriers’ if all 3 cultures were negative. Baseline *S. aureus* GIA results stratified by carriage status are shown for whole blood (**a**) and PBMC (**b**). Stratified using a reclassification of carriage types into two categories (‘persistent’ carriers and ‘others’) proposed by Van Belkum et al.^43^, *S. aureus* GIA baseline results are shown for whole blood (**c**) and PBMC (**d**). Bars represent the median values with the interquartile range (IQR). A Mann–Whitney test was performed where *indicates a *p*-value of < 0.05 and **indicates a *p*-value of < 0.005.

Associations were explored between *S. aureus* carriage and bacterial growth in both the whole blood and PBMC GIAs at baseline only, to avoid any confounding effects of BCG vaccination and Hb at follow-up time-points. Due to logistical limitations, *S. aureus* whole blood GIAs were performed on whole blood taken from volunteers enrolled into phase 1 only, while PBMC GIAs were performed on cryopreserved PBMC taken from volunteers enrolled into both phase 1 and phase 2. There was significantly higher growth of *S. aureus* at baseline in persistent carriers compared with non-carriers using the whole blood GIA (*p* = 0.01, Fig. 5a) which remained significant following correction for multiple comparisons (*p* = 0.02). Using the PBMC GIA, there was a trend towards higher growth in persistent carriers at baseline, although this was not statistically significant (Fig. 5b). Given that intermittent- and non-carriers share similar *S. aureus* nasal elimination kinetics and anti-staphylococcal antibody profiles, we also applied the binary reclassification of Van Belkum et al.^35^. Growth of *S. aureus* was significantly higher in persistent carriers compared with ‘others’ in whole blood (*p* = 0.007, Fig. 5c), with a non-significant trend in PBMC (Fig. 5d). There was no association between *S. aureus* carriage status and GIA outcome for the other bacteria under study using either classification system (Figure S3A-L).

## Discussion

In this randomised controlled study of BCG vaccination, we observed increased numbers of *M.tb* PPD-responsive IFN-γ secreting cells in the peripheral blood and improved control of mycobacterial growth in the direct PBMC MGIA following vaccination, which is compatible with previous findings and confirms vaccine ‘take’^36–39^. Interestingly, we saw an MGIA effect at 12 weeks but not 4 weeks post-BCG vaccination, which supports observations in other UK cohorts of a later peak MGIA response^36,40^. Using novel PBMC-based GIAs, we observed a significant reduction in the growth of the Gram-negative pathogens *E. coli* and *K. pneumoniae* at several early time-points following BCG vaccination which was not present in the control group. Such an early effect is consistent with data showing a trained immunity phenotype in monocytes by 3 days after training^41^. The NSE of BCG vaccination have been reported to last for many years and future work will be important to assess if the effects that we observed are maintained beyond 84 days. Our findings support a causal link between BCG vaccination and the *in-vitro* assay results, but elucidating the mechanism(s) concerned is beyond the scope of this study. We did not observe such effects with two Gram-positive pathogens, *S. aureus* and *S. agalactiae*. Although there were no significant differences between vaccinated and unvaccinated volunteers for any pathogen at any of the time-points studies, the power of this unpaired analysis was limited by the heterogeneity of responses and relatively small size of the control group which was a weakness of the study design.

Another finding, of importance when designing future GIAs and studies, concerns fluctuations in whole blood GIA responses in both groups, which was clear because of the randomised design and inclusion of an unvaccinated control group. This may have been driven by a batch effect due to real-time processing of fresh samples, or by changes in Hb concentration over time due to a relatively intensive bleed schedule, as both factors have been shown to influence the direct MGIA^36,42^. Using cryopreserved PBMC largely negated fluctuations in the control group. However, recent findings indicate a role for neutrophils in the NSE of BCG vaccination which would not be measured by a PBMC-based assay^43^.

The greatest strength of this study is its randomised experimental design which aids inference about the causal effects of vaccination. Other strengths include serial sampling of individuals, and our development of a novel assay designed to give a measure of functional bacterial control that is readily adaptable to a range of pathogens. Importantly, the PBMC-based GIA appears suitable for analysis of sequentially-collected samples which are later analysed under highly-standardised conditions. Weaknesses include a relatively small magnitude of effect under the assay conditions selected, and that we do not elucidate the immune parameters responsible for the observed association, although the GIA does include PBMC and autologous serum which permits assessment of the combined contributions of cellular and humoral responses. It is unclear whether the inter-individual variation observed is due to a poor assay signal-to-noise ratio or biological variability in volunteer responses, although previous *ex-vivo* studies have suggested the latter may be significant, supporting the need for a large sample size^21,23^. Furthermore, there was a logistical limit to the number of bacteria for which we could optimise GIAs, and it would be interesting to consider *S. pneumoniae* and SARS-CoV-2 in future studies, as well as the feasibility of GIAs for other pathogen types such as fungi and parasites which have been associated with NSE of BCG. Future studies could include a comparison with other whole-cell vaccines to determine whether the effect is particular to BCG.

These findings provide experimental support for NSE of BCG vaccination. Such an effect may explain the epidemiological studies and randomised trials indicating beneficial effects of BCG on reducing all-cause mortality^4–12^, and is consistent with murine studies demonstrating increased protection from *K. pneumoniae* challenge following administration of BCG or its components^44^. In the assay deployed, we only observed changes in the growth of Gram-negative pathogens (*E. coli* and *K. pneumonia*). Freyne et al.^45^ recently reported patterns of chemokine production in BCG-vaccinated infants that differed in response to Gram-positive and Gram-negative bacteria. Such ‘pathogen-specific’ in-vitro patterns of innate response following BCG vaccination may influence bacterial growth in our assays. Differential macrophage activation/phagocytosis, or the lack of whole blood factors such as neutrophils could account for this effect where they contribute differently to the control of Gram-positive and Gram-negative bacteria^46,47^. Some studies suggest more marked protective NSE of BCG in females than males^13–16,45^. While we observed a modest effect supportive of this in the *S. agalactiae* GIA, this was not the case for the other pathogens, possibly because our study was not powered to detect sex-specific effects.

The *in-vitro* effects observed in our GIAs in this cohort of North European volunteers are relatively modest. NSE of BCG have been primarily reported in African infants^4,6–8,13,48^, while our study was conducted on UK adults. Adults were chosen due to ethical and logistical feasibility, but these two populations are known to differ considerably in their specific response to BCG vaccination^2,49^, and may similarly differ in NSE and/or baseline pathogen exposure and common causes of mortality. Non-specific differences in cytokine production following BCG vaccination have also been noted between infants and adults^22,45^. As the NSE of BCG appear to decrease with age, we may have observed stronger effects in an infant population^50^. While we selected pathogens commonly associated with all-cause infant mortality, the list of pathogens potentially responsible for NSE of BCG is extensive including (but not limited to): respiratory syncytial virus, rotavirus, *Haemophilus influenza*, *Streptococcus pneumoniae*, *Salmonella typhi* and *paratyphi,* as well as malaria-causing *Plasmodium* spp. and fungal infections^3^. We, and others, observed NSE of differing magnitudes and kinetics in response to different pathogens, and selecting other pathogens may have altered outcomes.

A secondary aim of this study was to explore whether *S. aureus* nasal carriage influenced ability to control bacterial growth in-vitro. The anterior nares are a major reservoir of *S. aureus* in humans, and carriage is known to induce both innate and adaptive immune responses which we hypothesised may confound our GIA outcomes^51^. At any given time-point the prevalence of carriers was consistent with previous estimates in this population^52^. However, carriage status is dynamic and two categories of carrier have been described: persistent and intermittent. Nouwen et al.^53^ reported that the predictive value of two consecutive positive culture results for persistent carriage was 79%. We thus sampled at three time-points to discriminate between categories. Intermittent and persistent carriage rates were again similar to previous reports^53,54^. We observed increased growth of *S. aureus* in persistent carriers compared with non-carriers or ‘others’ using the whole blood GIA, with a similar trend in the PBMC GIA. A weaker effect in PBMC suggests the contribution of whole blood factors not present in PBMC such as neutrophils which play a critical role in acute inflammation and host defence against *S. aureus*^55^. There was no effect of carriage on growth of the other bacteria under study, suggesting a specific immunological mechanism.

While persistent *S. aureus* carriage is an unequivocal risk factor for infection, this is generally considered to be due to colonising strains serving as endogenous reservoirs for autoinfection^56^. However, consistent with our findings, Ghasemzadeh-Moghaddam et al*.*^57^ reported that nasal carriers were more likely to acquire exogenous *S. aureus* strains than non-carriers. Indeed, persistent carriers have by definition failed to prevent nasal colonisation, and secretions taken from carriers have been shown to be less damaging to *S. aureus*^58–60^. Our study has the limitations of inconsistency in positive cultures across all three swabs for volunteers classified as persistent carriers; applying the ‘culture rule’ definition based on quantitative as well as qualitative results of consecutive swabs would be preferable^53^. Sensitivity to detect low-level carriers may also have been reduced as swabs were not placed in enrichment broth prior to culture^61^. Nonetheless, a signal indicating an influence of *S. aureus* carriage on GIA outcome raises the possibility that our ability to observe BCG vaccine-induced NSE on *S. aureus* control was limited by this confounder. Colonisation status for other bacteria under study may also be relevant and this warrants further investigation.

In conclusion, we provide evidence in favour of NSE of BCG vaccination using *in-vitro* sum-of-the-parts functional PBMC GIAs for unrelated bacteria as a surrogate of protection. Such assays are potentially useful tools for the assessment of non-specific as well as specific effects of TB vaccines, although further development to maximise sensitivity and better understand the influence of colonisation status may be required. The assay also requires optimisation for each pathogen, for example in terms of multiplicity of infection, due to differing bacterial growth kinetics. Nonetheless, GIAs represent a tractable model that may be applied in future to investigate the immune mechanisms underlying NSE. An improved understanding of the NSE of BCG vaccination will be critical in directing the design of novel TB vaccines, and may be of value in the early control of future emerging pandemic pathogens.

## Materials and methods

### Study design and participants

A randomised controlled clinical study was conducted of healthy BCG-naïve, UK adults aged 18–45 years; see Table 1 for demographic data (Table 1). The study was reviewed and approved by the NHS Research Ethics Service (NRES) Committee South Central—Oxford B (REC reference 15/SC/0022) and registered with ClinicalTrials.gov (NCT02380508, 05/03/2015). It was conducted according to the principles of the Declaration of Helsinki and Good Clinical Practice. Eligibility criteria were: age 18–45, BCG naïve, resident in or near Oxford or Birmingham for the duration of the trial, no relevant findings in medical history or on physical examination, permission provided for investigators to discuss medical history with GP and to register volunteer details with a confidential database to prevent concurrent entry into other clinical studies, use of effective contraception for the duration of the study period (females), agreement to refrain from blood donation for the duration of the study period and for a period of three months after the final visit, and ability and willingness to comply with all the study requirements. Volunteers provided written informed consent prior to screening. Baseline biochemical and haematological analysis and serological testing for human immunodeficiency virus, hepatitis B and C virus were performed to ensure no abnormalities warranting exclusion. Latent TB infection (LTBI) was excluded at screening by T Spot.TB (Oxford Immunotec, UK) or QuantiFERON®-TB Gold In-Tube test using manufacturer-recommended cut-offs.

Volunteers were randomised to receive BCG SSI intradermally at a standard dose (2–8 × 10^5^ CFU) (*n* = 27) or to be unvaccinated controls who received no intervention (*n* = 8). Sample size was based on power to observe specific effects of BCG vaccination by IFN-γ ELISpot and MGIA. Volunteers were enrolled in two phases and in cohorts of 3–5 volunteers for logistical purposes (Fig. 1a) at either the Centre for Clinical Vaccinology and Tropical Medicine, Oxford, or the NIHR Wellcome Trust Clinical Research Facility, Birmingham. Block randomisation of 4:1, 3:1 or 2:1 vaccinated:control volunteers was used with block size determined by number of volunteers per enrolment schedule for that day. The mechanism used to implement the random allocation sequence was sealed envelopes prepared by an independent colleague at the Centre for Statistics in Medicine and opened only on the day of vaccination. Due to the global BCG shortage, volunteers in each phase were vaccinated with different batches of BCG SSI. At day 0 venepuncture was performed for GIA. Subsequent follow-up visits for venepuncture were at days 2, 4, 7, 10, 14, 21, 28 and 84; volunteers from Birmingham did not attend day 4 and 10 visits for logistical reasons. Nasal swabs were taken at screening, day 0 and 14. Laboratory staff were blinded until the final volunteers from the final schedule of each phase had completed their last visit. The study ran from February 2015 to November 2016 and ended when the final volunteer completed 84 days of follow-up. The primary outcome was ability to control of the growth of *S. aureus*, *K. pneumoniae*, *S. agalactiae* and *E. coli in-vitro* as measured by GIAs conducted at baseline and days 2, 4, 7, 10, 14, 21, 28 and 84 post-BCG vaccination*.* The study schedule is shown in Fig. 1b.

### Ex-vivo IFN-γ ELISpot

ELISpots were performed using a human IFN-γ ELISpot kit (capture mAb D1K, Mabtech). Triplicate wells containing 3 × 10^5^ fresh PBMC were stimulated for 18–20 h with purified protein derivative (PPD) from Statens Serum Institut (SSI) at a concentration of 20 μg/ml. Staphylococcal enterotoxin B (Sigma) was used as a positive control at a concentration of 10 μg/ml and unstimulated PBMCs were used to measure background IFN-γ production. Results are reported as spot forming cells (SFC) per million PBMC, calculated by subtracting the mean count of the unstimulated wells from the mean count of triplicate PPD-stimulated wells and correcting for the number of PBMC in the well.

### Direct PBMC mycobacterial growth inhibition assay (MGIA)

The PBMC MGIA was performed as previously described^39^. Briefly, 3 × 10^6^ PBMC and 500 CFU BCG Pasteur in a volume of 480 μl RPMI (containing 2 mM l-glutamine and 25 mM HEPES), plus 120 μl autologous serum per well were added to a 48-well plate. Co-cultures were incubated at 37 °C, 5% C0_2_ for 96 h, after which lysates were transferred to BACTEC MGIT tubes supplemented with PANTA antibiotics and OADC enrichment broth (Becton Dickinson, UK) for BCG quantification. Results are presented as normalised BCG growth which is equal to log_10_ CFU of sample minus log_10_ CFU of a growth control inoculated at the beginning of the assay.

### Bacterial growth inhibition assays (GIAs)

#### Preparation of bacterial cultures

*Staphylococcus aureus* wild-type Newman Strain was obtained from the American Type Culture Collection (ATCC). Wild-type *E. coli*, *K. pneumoniae*, and *S. agalactiae* clinical strains were isolated from blood cultures of human patients. Frozen stocks of a single colony were made. A scraping of cryopreserved stock was plated onto Blood Agar Base 2 with Horse Blood (Oxoid). Plates were incubated for 12–24 h at 37 °C and then plates were stored at 4 °C for up to 2 weeks until colonies were picked for the preparation of liquid media culture. The day before blood collection/PBMC sample thawing, three well-separated typical bacterial colonies were picked using a 10 µl loop and inoculated into 10 ml of fresh sterile liquid media; *S. aureus* and *S. agalactiae* were cultured in tryptic soy broth (Oxoid) while *E. coli* and *K. pneumoniae* were cultured in lysogeny broth (Sigma Aldrich). Bacteria were incubated for 14 h overnight at 37 °C in an orbital warm air incubator (130 rpm for *S. aureus*, 225–250 rpm for all other bacteria). Following overnight incubation, 100 µL of bacterial culture was inoculated into 10 ml of fresh media, followed by a second incubation period (1.5 h for *K. pneumoniae*, 2 h for *E. coli*, 2.5 h for *S. aureus* and 3 h for *S. agalactia*e). Bacteria were then centrifuged for 10 min at 3750 rpm. The supernatant was discarded, and the bacterial pellet resuspended and washed twice in 10 ml of phosphate buffered saline (PBS). The final pellet was resuspended in 10 ml of RPMI with Hepes modification (Gibco) and 1% L-glutamine (Gibco), and serial tenfold dilutions prepared in the same medium.

#### Whole blood GIAs

300 µl of heparinised blood was inoculated with 300 µl of bacterial inoculum at a concentration of 1 × 10^7^ CFU/ml in duplicate. Samples were incubated for 1 h in an orbital warm air incubator (130 rpm for *S. aureus*; 225–250 rpm for all other bacteria). Following centrifugation for 5 min at 12,000 rpm, supernatants were removed and the pellets lysed in 1 ml of sterile water for 10 min with a pulse-vortex at 0, 5 and 10 min. Following centrifugation for 10 min at 12,000 rpm, supernatants were removed and pellets resuspended in 1 ml PBS for quantification.

#### PBMC GIAs

PBMCs were thawed as previously described^39^ and prepared to a concentration of 1 × 10^6^ cells per 300 µl. 300 µl was added in duplicate to 48 well tissue culture plates. Following optimisation, the bacterial concentrations used were: 1 × 10^5^ CFU/ml for *S. aureus*, 3 × 10^4^ CFU/ml for *S. agalactiae*, and 2–4 × 10^3^ CFU/ml for *E. coli* and *K. pneumoniae*. 300 µL of bacterial inoculum was added to each well and incubated for 1 h at 37 °C, 5% CO_2_. Samples were transferred into 2 ml screw-cap tubes (Sarstedt) and centrifuged for 10 min at 12,000 rpm during which time 500 µl of sterile water was added to each well. Supernatants were removed and the water transferred to the tubes from the corresponding wells. Pellets were re-suspended by pulse vortexing.

#### Bacterial quantification

Samples were diluted 1:10 and 100 µL was plated onto blood agar base no. 2 with horse blood (Oxoid) using a spiral plater (Autoplate, Spiral Biotech). Plates were incubated overnight at 37 °C and counted the following day using an automated plate reader and software (QCount). Results are presented as the growth ratio which is equal to log_10_(CFU of sample/CFU of growth control).

### Determination of *S. aureus* carriage status

A sterile cotton bud (Technical Service Consultants Ltd.) was passed thoroughly around the rims of both anterior nares and placed into 2 ml of sterile PBS (Sigma). Samples were stored at 4 °C and processed within 24 h. 100 µl of sample was plated using an automated spiral plater (Autoplate, Spiral Biotech) onto *S. aureus* selective Staph Brilliance 24 agar plates (Oxoid) and incubated overnight at 37 °C, before quantification of dark blue colonies the following day.

### Statistical analysis

Data were analysed using GraphPad Prism v.8 and SPSS v.27. Normality was determined by Shapiro–Wilk. Comparisons between time-points were made using a paired t-test or Wilcoxon for normally or non-normally distributed data, respectively. Results are also reported following correction for multiple comparisons; a repeated measures one-way analysis of variance (RM-ANOVA), or mixed effects analysis if random values were missing, with Dunnett’s multiple comparisons test (all time-points vs. baseline) was performed for normally distributed data. Comparisons of GIA outcomes stratified by sex or *S. aureus* carriage status were made using a Mann–Whitney test and Kruskal–Wallis with Dunn’s correction for multiple comparisons.

### Conference presentation

This work was presented in part at the 27th European Congress of Clinical Microbiology and Infectious Diseases, April 2017 in Vienna, Austria.

## Supplementary Information

Supplementary Information.

## Data Availability

The datasets generated during and/or analysed during the current study are available from the corresponding author on reasonable request.
