# Supplementary material for: Functional in-vitro evaluation of the non-specific effects of BCG vaccination in a randomised controlled clinical study
Source: Sci Rep. 2022 May 12;12:7808. doi: 10.1038/s41598-022-11748-x (PMC9096342; doi:10.1038/s41598-022-11748-x)
Supplement: Supplementary file 1 — Supplementary Information. [file 41598_2022_11748_MOESM1_ESM.pdf]

Supplementary Material

Functional *in-vitro* evaluation of the non-specific effects of BCG vaccination in a randomised controlled clinical study

Running title: Non-specific effects of BCG vaccination

Morven Wilkie ϕ, Jenner Institute, Nuffield Department of Medicine, University of Oxford, Oxford, OX3 7DQ, UK.

Rachel Tanner ϕ, Jenner Institute, Nuffield Department of Medicine, University of Oxford, Oxford, OX3 7DQ, UK. Telephone: 01865617615, Email: rachel.tanner@ndm.ox.ac.uk

Daniel Wright, Jenner Institute, Nuffield Department of Medicine, University of Oxford, Oxford, OX3 7DQ, UK.

Raquel Lopez-Ramon, Jenner Institute, Nuffield Department of Medicine, University of Oxford, Oxford, OX3 7DQ, UK.

Julia Beglov, Jenner Institute, Nuffield Department of Medicine, University of Oxford, Oxford, OX3 7DQ, UK.

Michael Riste, Jenner Institute, Nuffield Department of Medicine, University of Oxford, Oxford, OX3 7DQ, UK; Department of Infection and Tropical Medicine, Birmingham Heartlands Hospital, Birmingham, B9 5SS, UK.

Julia L. Marshall, Jenner Institute, Nuffield Department of Medicine, University of Oxford, Oxford, OX3 7DQ, UK.

Stephanie A. Harris, Jenner Institute, Nuffield Department of Medicine, University of Oxford, Oxford, OX3 7DQ, UK.

Paulo Bettencourt, Jenner Institute, Nuffield Department of Medicine, University of Oxford, Oxford, OX3 7DQ, UK.

Ali Hamidi, Jenner Institute, Nuffield Department of Medicine, University of Oxford, Oxford, OX3 7DQ, UK.

Pauline M. van Diemen, Jenner Institute, Nuffield Department of Medicine, University of Oxford, Oxford, OX3 7DQ, UK.

Paul Moss, Department of Infection and Tropical Medicine, Birmingham Heartlands Hospital, Birmingham, B9 5SS, UK.

Iman Satti, Jenner Institute, Nuffield Department of Medicine, University of Oxford, Oxford, OX3 7DQ, UK.

David Wyllie, Jenner Institute, Nuffield Department of Medicine, University of Oxford, Oxford, OX3 7DQ, UK.

Helen McShane*, Jenner Institute, Nuffield Department of Medicine, University of Oxford, Oxford, OX3 7DQ, UK.

* Corresponding author

ϕ Contributed equally

	D0	D2	D7	D14	D28	D84
S. aureus	p=0.15	p=0.23	p=0.69	p=0.23	p=0.42	p=0.88
S. agalactiae	P=0.57	**p=0.03***	p=0.18	p=0.57	p=0.57	p=0.09
E. coli	p=0.87	p=0.55	p=0.67	p=0.31	p=0.27	p=0.92
K. pneuomiae	p=0.23	p=0.27	p=0.27	p=0.44	p=0.44	p=0.39

Table S1. Differences in bacterial growth control stratified by sex. Growth of *S. aureus*, *S. agalactiae*, *E. coli* and *K. pneumoniae* in the PBMC GIAs was stratified by sex and a Mann-Whitney test performed comparing males and females at each time-point.

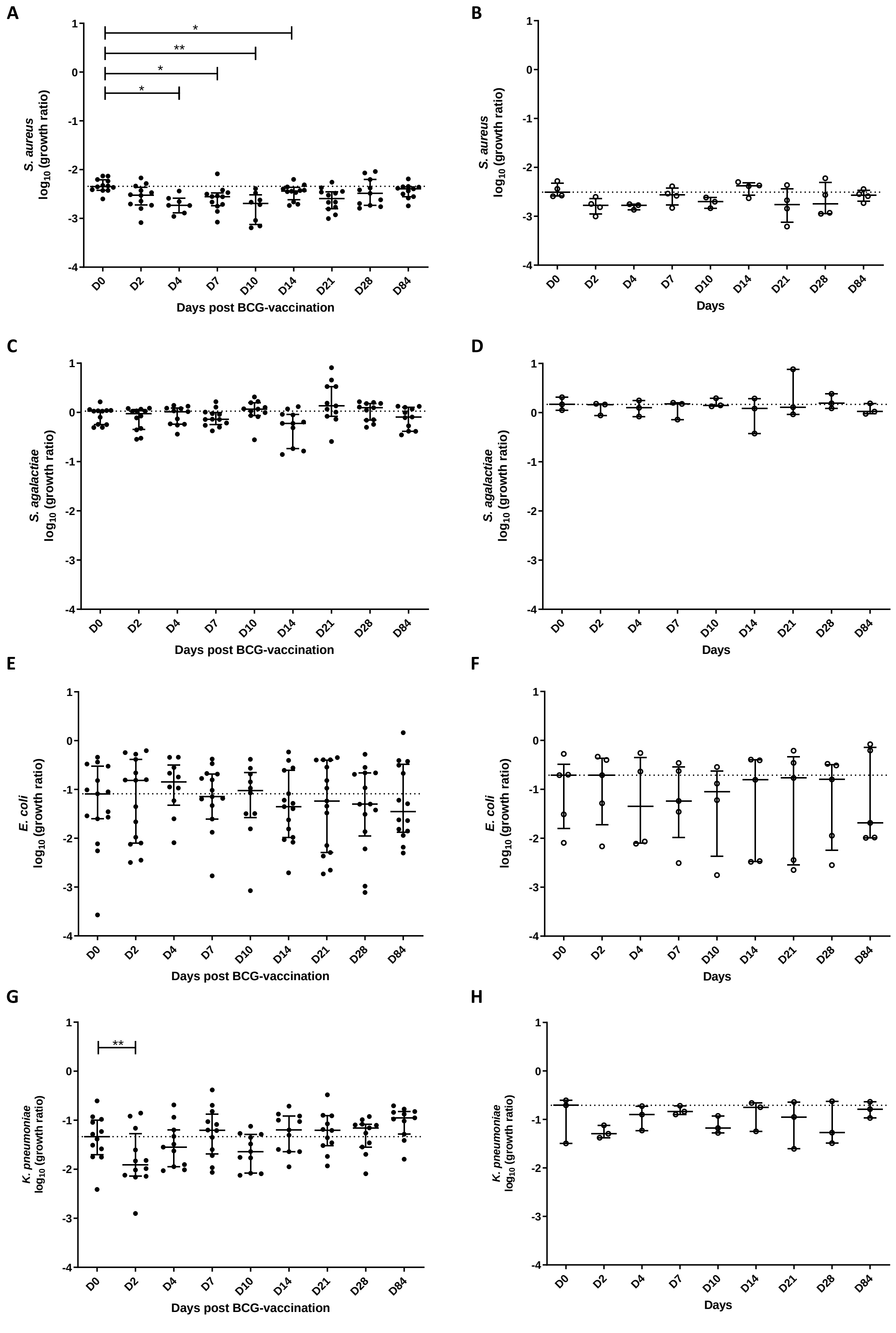
A
B
C
D
E
F
G
H
S. aureus log10 (growth ratio)
S. agalactiae log10 (growth ratio)
E. coli log10 (growth ratio)
K. pneumoniae log10 (growth ratio)
D0
D2
D4
D7
D10
D14
D21
D28
D84
Days post BCG-vaccination
Days
*
**

Figure S1. Whole blood growth inhibition assays suffered from batch effects. Healthy UK adults enrolled into phase 1 were randomised to receive BCG vaccination (n=15) or to be unvaccinated controls (n=5) and GIAs were conducted using whole blood co-cultured with *S. aureus* (**A-B**) or *E. coli* (**E-F**). Volunteers enrolled into phase 2 were also randomised to receive BCG vaccination (n=12) or to be unvaccinated controls (n=3) and GIAs were conducted using whole blood co-cultured with *S. agalactiae* (**C-D**) or *K. pneumoniae* (**G-H**). After 1 hour, cells were lysed and bacteria quantified by plating on solid blood agar. Whole blood GIAs were conducted on samples taken at baseline and days 2, 4, 7, 10, 14, 21, 28 and 84 following BCG vaccination (closed circles) and at the same time-points in unvaccinated control individuals (open circles). Bars represent the median values with the IQR; dotted lines indicate the median at baseline. Paired t-tests were performed of each time-point vs. baseline, where * indicates a p-value of <0.05 and ** indicates a p-values of <0.005.

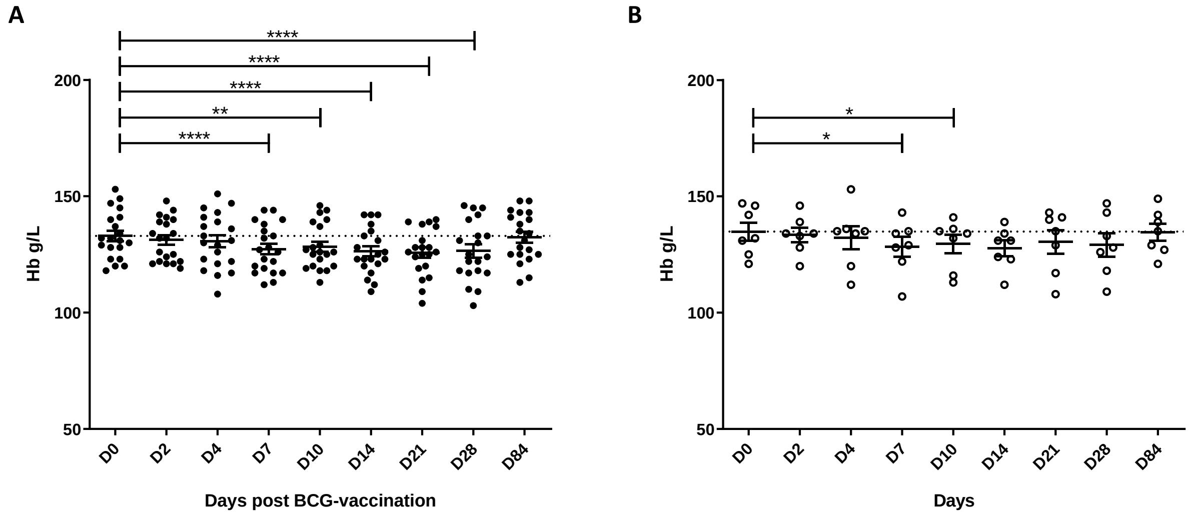

Figure S2. Haemoglobin concentration decreases over time. The haemoglobin (Hb) concentration was measured at each time-point in volunteers that received BCG vaccination (**A**) and unvaccinated control individuals (**B**). Paired t-tests were performed of each time-point vs. baseline, where * indicates a p-value of <0.05, ** indicates a p-value of <0.005 and

**** indicates a p-value of <0.0001. Bars represent the mean values with the SEM; dotted lines indicate the baseline mean.

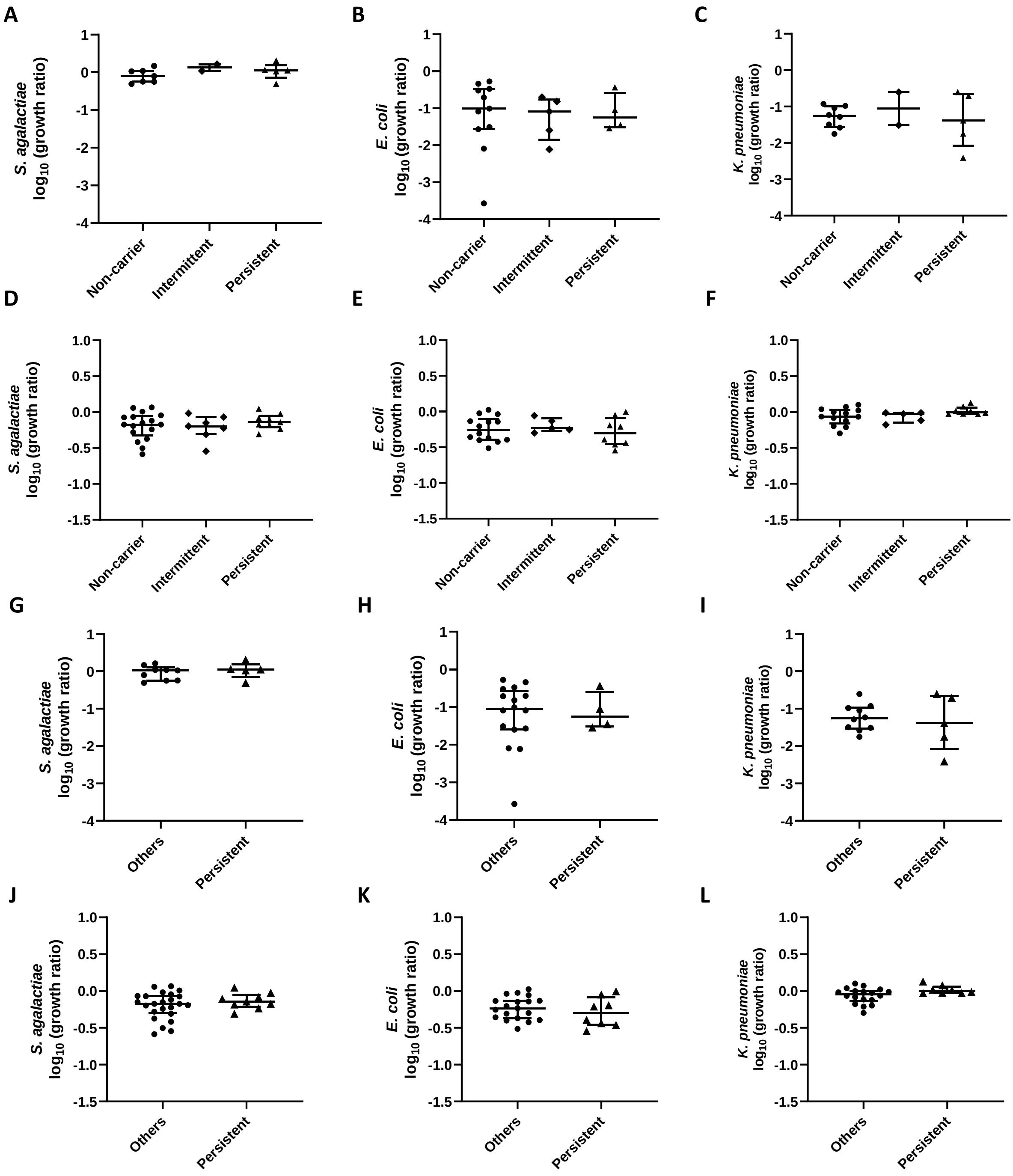

Figure S3. Growth of other bacteria in the GIA stratified by *S.aureus* carriage status. The anterior nares of all volunteers were sampled at screening and days 0 and 14. Individuals were considered ‘persistent’ carriers if two or more consecutive cultures were

positive, ‘intermittent’ carriers if one or more non-consecutive cultures were positive or ‘non-carriers’ if all 3 cultures were negative. Baseline GIA results for *S. agalactiae, E. coli* and *K. pneumoniae* stratified by carriage status are shown for whole blood (**A-C** respectively) and PBMC (**D-F** respectively). Stratified using a reclassification of carriage types into two categories (‘persistent’ carriers and ‘others’) proposed by Van Belkum *et al*. [43], *S. agalactiae, E. coli* and *K. pneumoniae* GIA results are shown baseline for whole blood (**G-I** respectively) and PBMC (**J-L** respectively). Bars represent the median values with the interquartile range (IQR).
